# The impact of follow-up blood cultures on mortality and management in patients with gram-negative bloodstream infections: a validation cohort study

**DOI:** 10.1186/s12879-026-13579-x

**Published:** 2026-05-28

**Authors:** Joshua T. Thaden, Felicia Ruffin, Lawrence P. Park, Parisa Farahani, Divyam Goel, Joshua B. Parsons, Vance G. Fowler, Stacey A. Maskarinec

**Affiliations:** 1 Division of Infectious Diseases, Department of Medicine, Duke University, DUMC Box 102359, Durham, NC 27710 USA; 2 Carillion Clinic, 213 South Jefferson St. SW, 11th Floor, Roanoke, VA 24011 USA; 3 Duke University School of Medicine, DUMC Box 102359, Durham, NC 27710 USA; 4 Duke Clinical Research Institute, DUMC Box 102359, Durham, NC 27710 USA

**Keywords:** Gram negative, Bloodstream infection, Bacteremia, Follow-up blood culture

## Abstract

**Background:**

The practice of obtaining follow-up blood cultures (FUBCs) in patients with gram-negative bloodstream infections (GN-BSI) remains controversial. The current study had two goals: (1) to internally validate our earlier findings of decreased mortality in patients with FUBCs and increased mortality in those with persistent GN-BSI, and (2) to identify mechanisms by which FUBCs might impact clinical care and outcome.

**Methods:**

Adults with GN-BSI at Duke University were enrolled from 2015 to 2021. Cox proportional hazards models with propensity score-weighting were used to examine associations of FUBCs and positive FUBCs with attributable and all-cause in-hospital mortality. Immortal time bias was addressed by treating FUBCs as a time-dependent variable and through sensitivity analyses. To understand the potential impact of FUBCs on clinical care, matched cohorts were generated (*n* = 41 in each group): (1) no FUBCs, (2) FUBCs negative for growth, and (3) FUBCs positive for growth (i.e., persistent GN-BSI). Infection-related consultations, imaging studies, and procedures were determined for each matched cohort.

**Results:**

Of the 1291 patients in this study, FUBCs were obtained in 78% (1012 patients) and positive in 20% (199 patients). After adjusting for immortal time bias, obtaining FUBCs remained associated with decreased attributable mortality (Hazard ratio [HR] 0.39; 95% confidence interval [CI] 0.32–0.49; *p* < 0.0001). Persistent GN-BSI was associated with increased attributable mortality (HR 1.77; 95% CI, 1.24–2.52; *p* = 0.002). Obtaining FUBCs was associated with subsequent infectious diseases (ID) consultation (No FUBCs: 2/41 [5%]; FUBCs: 21/82 [26%]; *p* < 0.0001). This finding was primarily due to patients with persistent GN-BSI (FUBCs negative: 6/41 [15%]; FUBCs positive: 15/41 [37%]; *p* = 0.04). Patients with persistent GN-BSI had a higher rate of source control procedures (FUBCs positive: 12/41 [29%]; All others: 11/82 [13%]); *p* = 0.05).

**Conclusions:**

Obtaining FUBCs was associated with decreased mortality. This mortality effect could stem from increased ID consults after FUBCs and increased source control procedures following positive FUBCs.

**Supplementary Information:**

The online version contains supplementary material available at 10.1186/s12879-026-13579-x.

## Background

Gram-negative bloodstream infection (GN-BSI) is common and associated with high healthcare costs [1, 2] and mortality [3, 4]. Obtaining follow-up blood cultures (FUBCs) in patients with GN-BSI to document clearance of bacteremia is controversial. Using a cohort of patients with GN-BSI from 2002 to 2015 at Duke University, we previously showed that obtaining FUBCs was associated with decreased mortality [5]. Although this finding was confirmed by three meta-analyses [6–8], a subsequent large study found no such association [9]. Further, recent shortages of blood culture bottles have accentuated the need to establish the role of obtaining FUBCs on patients with GN-BSI [10]. In total, the impact of FUBCs on the outcome of patients with GN-BSI is unresolved.

In the current study, we used a large and more contemporary cohort (2015–2021) of prospectively enrolled patients with GN-BSI to pursue two goals: (1) internally validate the results of our previous publication [5]; and (2) better understand the impact of FUBCs on clinical management of patients with GN-BSI. To accomplish these goals, we created matched sets of patients from the overall cohort and compared rates of infection-related consultations, imaging studies, and procedures between the groups.

## Methods

### Overall study population

From 01 July 2015 through 31 December 2021, eligible adult inpatients with GN-BSI at Duke University Health System were prospectively enrolled. The enrollment process and criteria were similar to our prior study [5]. This study was approved by the Duke Institutional Review Board (IRB). Written informed consent was obtained from patients or their legal representative. In patients with multiple hospitalizations with GN-BSI over the study, only the first such hospitalization was included. Patients were excluded if they had polymicrobial bacteremia, enrolled in hospice, neutropenic, or died within 24 h of initial blood culture.

### Matched cohorts

**Fig. 1:**
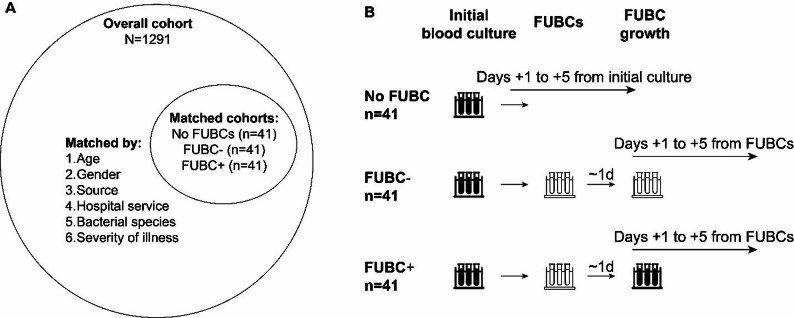
Study schematic. (**A**) Associations between follow-up blood cultures (FUBCs) and mortality were determined with the overall patient cohort (*N* = 1291). To address associations between FUBC and management decisions (e.g., consultations, imaging, procedures), matched cohorts of patients without FUBCs (*n* = 41), FUBCs obtained and negative for growth (FUBC-; *n* = 41), and FUBCs obtained and positive for growth with same bacterial species (FUBC+; *n* = 41) were generated from the overall cohort. (**B**) For patients in the matched cohorts, infection-related management decisions were then determined both after the initial positive blood culture generally and during particular time windows shown here. The time windows from + 1 to + 5 days after the FUBCs were selected to identify infection-related management decisions that could have been driven by the FUBC result. For patients without FUBCs, infection-related management decisions were determined from + 1 to + 5 days after the initial positive blood culture

To investigate the impact of FUBCs on clinical practice, we generated three matched cohorts from the overall cohort: those with (1) no FUBCs, (2) FUBCs obtained and negative for growth, and (3) FUBCs obtained and positive for growth (Fig. 1A). All patients in the matched cohorts had to have survived at least 7 days from their last FUBC (if they were obtained) or the initial positive blood culture (if FUBCs not obtained). Patients in the three cohorts were matched with respect to age, gender, source of GN-BSI, hospital service, bacterial species groups (i.e., Enterobacterales versus non-Enterobacterales), and severity of illness as determined by the Pitt bacteremia score [11]. For patients in the matched cohorts, the medical records were reviewed for infection-related consultations, imaging, and procedures. Consultations were deemed to be infection-related if the consult was obtained to address the evaluation and management of the GN-BSI. Imaging studies were deemed to be infection-related if they were ordered to assess the source or extent of the underlying GN-BSI. The results of the infection-related imaging studies were categorized as either consistent with infection (i.e., clearly indicated presence of infection), possible infection (i.e., equivocal imaging report), or inconsistent with infection (i.e., no evidence of infection). Procedures were deemed to be infection-related if they directly involved the diagnosis (e.g., arthrocentesis) or treatment (e.g., line removal) of the GN-BSI.

Infection-related consultations, imaging, and procedures were identified within two time windows. First, infection-related events were determined from the time of the initial positive blood culture to 7 days past the FUBC (when obtained). In cases where no FUBC was obtained, we identified infection-related events in the 7 days after the initial positive blood culture. Second, to identify events that may have been impacted by the FUBCs, we examined infection-related consultations, imaging studies, and procedures in a period from day 1 through day 5 following the FUBC (Fig. 1B). As a comparator, we similarly examined such instances from day 1 through day 5 after the initial blood culture in patients without FUBCs. The infection-related consultations, imaging, and procedures both during and after discharge were queried, though in practice no such events were identified after patient discharge within the time windows. Thus the infection-related investigations were completed within the index hospitalization.

### Definitions

A FUBC was defined as a blood culture drawn from 24 h to 7 days after the initial positive blood culture, consistent with our prior and other studies [5, 12]. Persistent GN-BSI is defined as a positive FUBC with the same organism. In-hospital mortality, which is death during the hospitalization associated with the GN-BSI episode, was determined. All deaths were categorized as attributable or non-attributable. Attributable mortality is defined as death secondary to GN-BSI and included all patients who died with persistent signs or symptoms of systemic infection, ongoing positive blood culture results, or a persistent focus of infection in the absence of another explanation for death. Attributable mortality was determined by members of the clinical research team after the event. Non-attributable mortality included cases of in-hospital mortality that did not meet the attributable mortality definition. Additional definitions are provided in the Supplemental Methods.

### Statistical analyses

The primary outcomes in this study were the all-cause and attributable in-hospital mortality associated with FUBCs and persistent GN-BSI. Secondary outcomes included infection-related consultations, imaging studies, and procedures in patients without FUBCs, FUBCs positive for growth (i.e., persistent GN-BSI), and FUBCs negative for growth. Baseline characteristics and clinical events are presented as means with standard deviation for continuous variables and frequencies with proportions for categorical variables. Statistical comparisons between groups for continuous variables were made with t-tests. For categorical variables, comparisons were made using Fisher’s exact tests (for binary comparisons) or chi-square tests (for > 2 groups). To adjust for selection bias between patients who did and did not have FUBCs drawn, a propensity score-based inverse probability weighting was performed. A logistic regression propensity score model predicting FUBCs was fit and used as an inverse weighting to balance the differences between the groups of patients with and without FUBCs. For evaluation of the performance of the propensity score models, variables were evaluated comparing means or proportions of the included variables by levels of FUBC drawn in unweighted and weighted analyses, and covariate balance and p-values were compared between them. After weighting by the propensity score, these groups did not differ meaningfully nor statistically on any of the covariables included in the outcome models. Covariates in these models included age, race, gender, medical comorbidities (transplant recipient, hemodialysis dependence, Charlson comorbidity index), corticosteroid use in last 30 days, source of bacteremia, route of bacteremia, presence of a cardiac device, presence of a central venous catheter, hospital service, bacterial species, and days to effective antibiotic therapy. These covariates were selected to be broadly representative of the clinical factors that may influence the decision on whether to acquire FUBCs. Inverse probabilities of obtaining an FUBC, trimmed at 20, were used as weights in Cox proportional hazards models predicting mortality. These models were used to assess the association of covariates with clinical outcomes of attributable and all-cause mortality. In the adjusted models for mortality, immortal time bias was addressed in two ways: (1) FUBCs were considered a time-varying variable, and (2) sensitivity analyses were performed to determine if excluding patients that died within 48 and 72 h from initial blood culture altered our findings. This propensity score-based inverse probability weighted Cox proportional hazards analysis method was similarly used to compare patients with positive versus negative FUBCs. Associations between infection-related consultations, imaging studies, and procedures with FUBC groups were determined with Fisher’s exact tests. A two-sided p-value less than 0.05 was considered significant. All analyses were performed using SAS 9.4 (SAS Institute, Cary, NC).

## Results

### Overall study cohort

A total of 1291 patients were included in this study. FUBCs were obtained in 1012 (78%) patients. The mean time to FUBC was 2.1 days (standard deviation 1.1 days) from the initial positive blood culture. Characteristics of patients with and without FUBCs are shown in Table 1. Patients with FUBCs were younger (mean 62 years [standard deviation 16 years ] vs. 67 years [16 years]; *p* < 0.0001) and more often had hematopoietic or solid organ transplant (171/1012 [17%] vs. 11/279 [4%]; *p* < 0.0001), recent treatment with corticosteroids (337/1012 [33%] vs. 59/279 [21%]; *p* < 0.0001), hemodialysis dependence (92/1012 [9%] vs. 14/279 [5%]; *p* = 0.03), a central venous catheter (307/1012 [30%] vs. 39/279 [14%]; *p* < 0.0001), and hospital-acquired GN-BSI (271/1012 [27%] vs. 46/279 [16%]; *p* < 0.0001). Obtaining FUBCs differed by hospital service (*p* = 0.005), with surgical providers more frequently obtaining FUBCs.

**Table 1:** Patients included in this study. Abbreviation: FUBCs, follow-up blood cultures; GN-BSI, gram-negative bloodstream infection. P-values < 0.05 are indicated in bold

Variable	FUBCs not obtained *N* = 279 *n* (%)	FUBCs obtained *N* = 1012 *n* (%)	*P*-value
**Age (mean [standard deviation])**	67 (16)	62 (16)	**< 0.0001**
**Female sex**	138 (49)	479 (47)	0.92
**Race**			0.09
White	176 (63)	603 (60)	
Black	82 (29)	357 (35)	
Other	21 (8)	52 (5)	
**Medical comorbidities**			
IV drug use	3 (1)	28 (3)	0.12
Corticosteroid use	59 (21)	337 (33)	**< 0.0001**
Hematopoietic or solid organ transplant	11 (4)	171 (17)	**< 0.0001**
Hemodialysis	14 (5)	92 (9)	**0.03**
**Charlson comorbidity index (mean [standard deviation])**	9.0 (4.1)	8.6 (4.0)	0.13
**Medical devices present**			
Central venous catheter	39 (14)	307 (30)	**< 0.0001**
Cardiac device	31 (11)	135 (13)	0.36
**Source of GN-BSI**			**< 0.0001**
Genitourinary	149 (53)	389 (38)	
Intra-abdominal	54 (19)	181 (18)	
Skin/soft tissue	16 (6)	78 (8)	
Endovascular	3 (1)	91 (9)	
Pulmonary	13 (5)	74 (7)	
Bone/joint	1 (< 1)	2 (< 1)	
Other	12 (4)	49 (5)	
None identified	31 (11)	148 (15)	
**Route of GN-BSI**			**< 0.0001**
Hospital-acquired	46 (16)	271 (27)	
Community-acquired, healthcare-associated	96 (34)	387 (38)	
Community-acquired, non-healthcare-associated	137 (49)	354 (35)	
**Hospital service**			**0.005**
Medicine	173 (62)	536 (53)	
Surgery	52 (19)	283 (28)	
Intensive care unit (ICU)	54 (19)	193 (19)	
**Pitt bacteremia score (mean [standard deviation])**	2.7 (2.8)	2.4 (2.5)	0.47
**Days to effective antibiotic therapy**			0.08
0	220 (79)	751 (74)	
1	46 (17)	174 (17)	
2	5 (2)	36 (4)	
≥3	6 (2)	50 (5)	
**Length of hospital stay following initial positive blood culture (mean [standard deviation])**	5.9 (9.2)	14.2 (21.8)	**< 0.0001**
**Bacterial genus**			**0.0006**
* Escherichia*	151 (54)	395 (39)	
* Klebsiella*	52 (19)	256 (25)	
* Pseudomonas*	23 (8)	97 (10)	
* Enterobacter*	11 (4)	70 (7)	
* Serratia*	13 (5)	50 (5)	
Other	29 (10)	144 (14)	

### Association of FUBCs with patient mortality

**Fig. 2:**
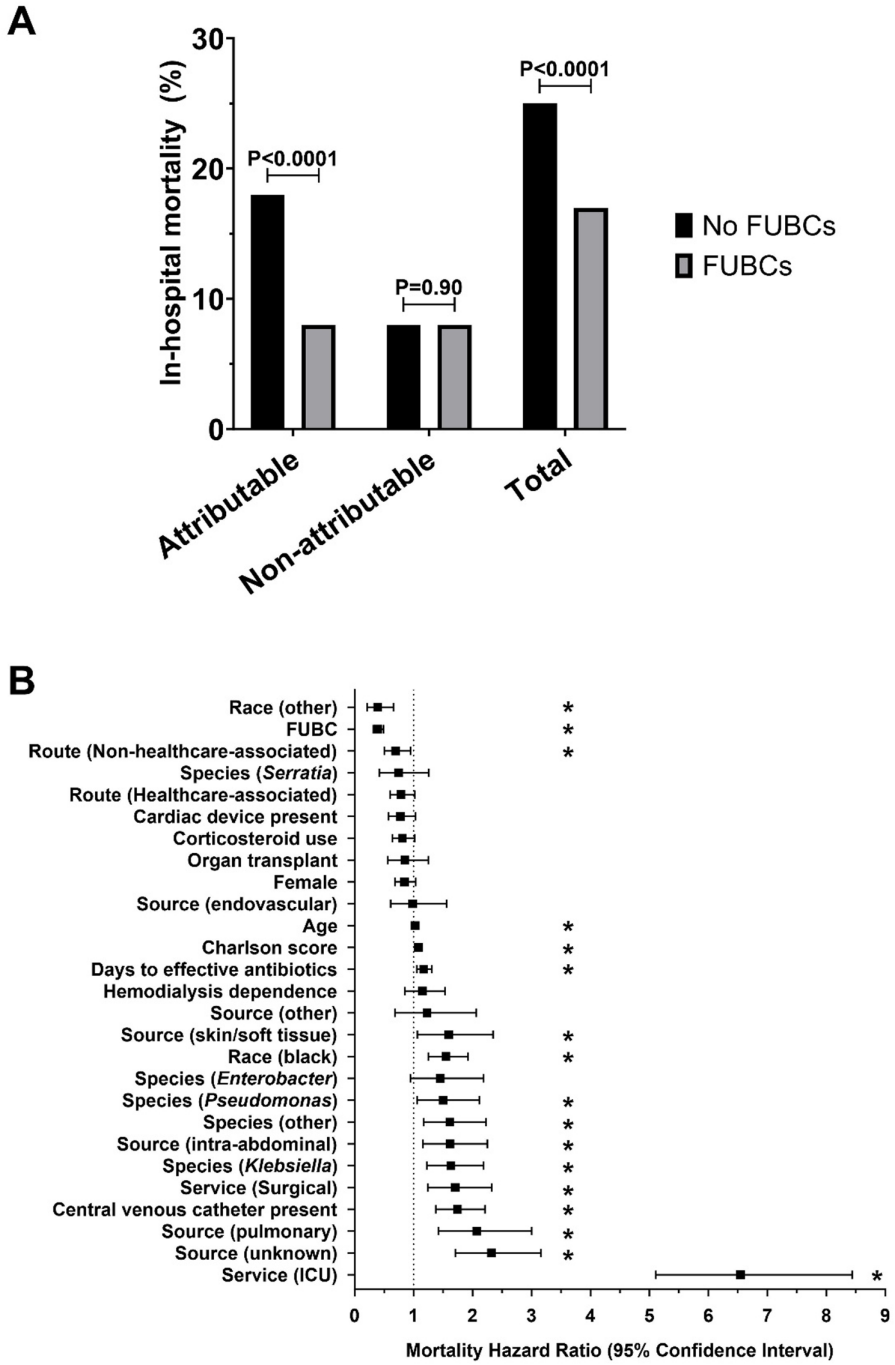
Association of follow-up blood cultures (FUBCs) with mortality in patients with gram-negative bloodstream infection (GN-BSI). (**A**) Unadjusted in-hospital attributable (i.e., death due to the infection), non-attributable (i.e., death due to other causes), and all-cause mortality is shown, stratified by whether FUBCs obtained. (**B**) Propensity score-weighted Cox regression model of attributable in-hospital mortality in patients with GN-BSI. Reference variables are as follows: Bacterial species, *Escherichia* species; Hospital service, Medicine; Race, White; Route, Hospital-acquisition; Source, Genitourinary. Variables with p-value less than 0.05 are indicated with an asterisk (*)

In-hospital attributable mortality, non-attributable mortality, and all-cause mortality were 10% (132/1291), 8% (106/1291), and 18% (238/1291), respectively. In an unadjusted analysis, obtaining FUBCs was associated with decreased attributable mortality (FUBCs: 83/1012 [8%]; no FUBCs: 49/279 [17%]; *p* < 0.0001) and all-cause mortality (FUBCs: 167/1012 [17%]; no FUBCs: 71/279 [25%]; *p* = 0.0009), but not with non-attributable mortality (FUBCs: 84/1012 [8%]; no FUBCs: 22/279 [8%]; *p* = 0.90) (Fig. 2A). An unadjusted survival curve for patients with and without FUBCs is shown in Supplemental Fig. 1. A propensity score-weighted Cox proportional hazards regression model was used to evaluate differences in clinical outcomes in GN-BSI patients with and without FUBCs. Obtaining FUBCs was associated with lower rates of both attributable (Hazard ratio [HR] 0.39; 95% confidence interval [CI] 0.32–0.49; *p* < 0.0001) (Fig. 2B) and all-cause mortality (HR 0.57; 95% CI 0.48–0.67; *p* < 0.0001) (Supplemental Fig. 2).

In the adjusted models of mortality, immortal time bias was addressed in two ways: FUBCs were considered a time-varying variable in the Cox proportional hazards models; and sensitivity analyses were performed to determine if excluding patients that died within 48–72 h from the initial positive blood culture altered our findings. FUBCs remained associated with decreased attributable mortality when patients that died within 48 h (HR 0.55; 95% CI 0.43–0.70; *p* < 0.0001) and within 72 h (HR 0.54; 95% CI 0.42–0.71; *p* < 0.0001) were excluded. FUBCs remained associated with decreased all-cause mortality when patients that died within 48 h were excluded (HR 0.83 95% CI 0.69–0.998; *p* = 0.048), though not when patients that died within 72 h were excluded (HR 0.88; 95% CI 0.72–1.08; *p* = 0.21) (Supplemental Fig. 3).

### Association of persistent GN-BSI with patient mortality

**Fig. 3:**
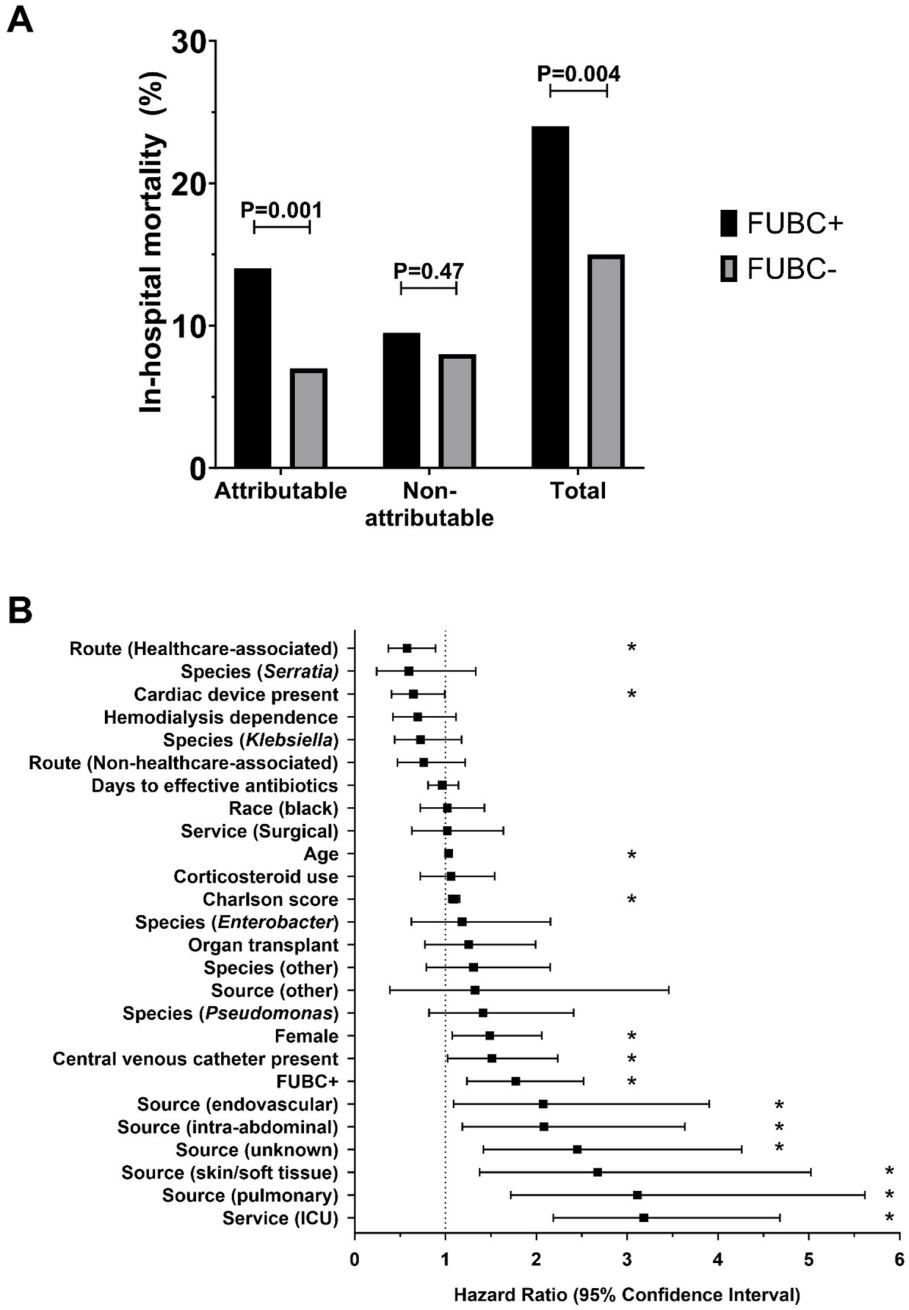
Association of persistent gram-negative bloodstream infection (GN-BSI) with mortality. (**A**) Unadjusted in-hospital attributable (i.e., death due to the infection), non-attributable (i.e., death due to other causes), and all-cause mortality is shown, stratified by whether the follow-up blood culture was positive (FUBC+) or negative (FUBC-). (**B**) Propensity score-weighted Cox regression model of attributable in-hospital mortality in patients with GN-BSI and FUBCs. Reference variables are as follows: Bacterial species, *Escherichia* species; Hospital service, Medicine; Race, White; Route, Hospital-acquisition; Source, Genitourinary. Variables with p-value less than 0.05 are indicated with an asterisk (*)

Among patients with FUBCs (*n* = 1012), 199 (20%) had positive FUBCs (i.e., persistent GN-BSI). Patients with persistent GN-BSI, relative to those with negative FUBCs, exhibited increased attributable (28/199 [14%] vs. 55/813 [7%]; *p* = 0.001) and all-cause mortality (47/199 [24%] vs. 120/813 [15%], *p* = 0.004), but not non-attributable mortality (19/199 [10%] vs. 65/813 [8%], *p* = 0.47) (Fig. 3A). A propensity score-weighted multivariable Cox proportional hazards analysis showed that persistent GN-BSI, relative to negative FUBCs, was associated with increased attributable mortality (HR 1.77; 95% CI, 1.24–2.52; *p* = 0.002) (Fig. 3B) but not all-cause mortality (HR 1.20; 95% CI, 0.92–1.55; *p* = 0.17) (Supplemental Fig. 4).

### Differences in clinical management based on FUBC acquisition status with matched cohort

To better understand the mechanisms of FUBC-associated variation in patient mortality, we generated three matched patient cohorts that included patients with: (1) No FUBCs, (2) FUBCs obtained and negative for growth, and (3) FUBCs obtained and positive for growth (Fig. 1). Characteristics of patients in the matched cohorts are shown in Supplemental Table 1. To understand how general clinical care differs between these cohorts, we first identified all infection-related consultations, imaging studies, and procedures from the initial positive blood culture through seven days past either the FUBC (if obtained) or the initial positive blood culture (if no FUBC obtained). These infection-related events may have occurred before or after obtaining FUBCs and therefore are not necessarily temporally associated with FUBCs.

In the 123 patients included in the matched cohorts, there were 76 infection-related consultations in 59 (59/123 [48%]) patients (Supplemental Table 2). The most common consultations included infectious diseases (ID) (39/76 [51%]) and urology (19/76 [25%]). Consultation rate was highest in patients with FUBCs (No FUBCs: 8/41 [20%]; FUBCs: 51/82 [62%]; *p* < 0.0001) (Supplemental Fig. 5A). Stratification of consultations by ID vs. non-ID revealed that the increased consultations in patients with FUBCs was secondary to a higher number of ID consultations in this group (No FUBCs: 3/41 [7%] ID consults; FUBCs: 36/82 [44%] ID consults; *p* = 0.003) (Supplemental Fig. 5B).

In the 123 patients included in the matched cohorts, there were 150 infection-related imaging studies performed in 81 (81/123 [66%]) patients. The most performed studies were computed tomography (CT) scans (62/150 [41%]) and x-rays (36/150 [24%]) (Supplemental Table 2). Patients with FUBCs more often had infection-related imaging studies (No FUBCs: 19/41 [46%]; FUBCs: 65/82 [79%]; *p* = 0.001) (Supplemental Fig. 5C). Among the 150 infection-related imaging studies, 39 (26%) were consistent with infection, 32 (21%) showed possible infection, and 79 (53%) were inconsistent with infection (Supplemental Table 3). When we restricted to imaging studies that were consistent with infection or possible infection, there was no association between imaging studies and obtaining FUBCs (No FUBCs: 15/41 [37%]; FUBCs: 29/82 [35%]; *p* = 0.34) or positive FUBCs (Negative FUBCs: 21/41 [51%]; Positive FUBCs: 18/41 [44%]; *p* = 0.66).

In the 123 patients included in the matched cohorts, there were 38 infection-related procedures performed with 33 patients (33/123 [27%]). The most common procedures were percutaneous drain placement (9/38 [24%]), endoscopic retrograde cholangiopancreatography (ERCP)/cholangiography (6/38 [16%]), and line removal (6/38 [16%]). Positive FUBCs, relative to all others, were associated with numerically increased procedures though no statistical significance (FUBCs positive 16/41 [39%]; all others 17/82 [21%]; *p* = 0.051) (Supplemental Fig. 5D).

### Differences in clinical management after FUBC

**Fig. 4:**
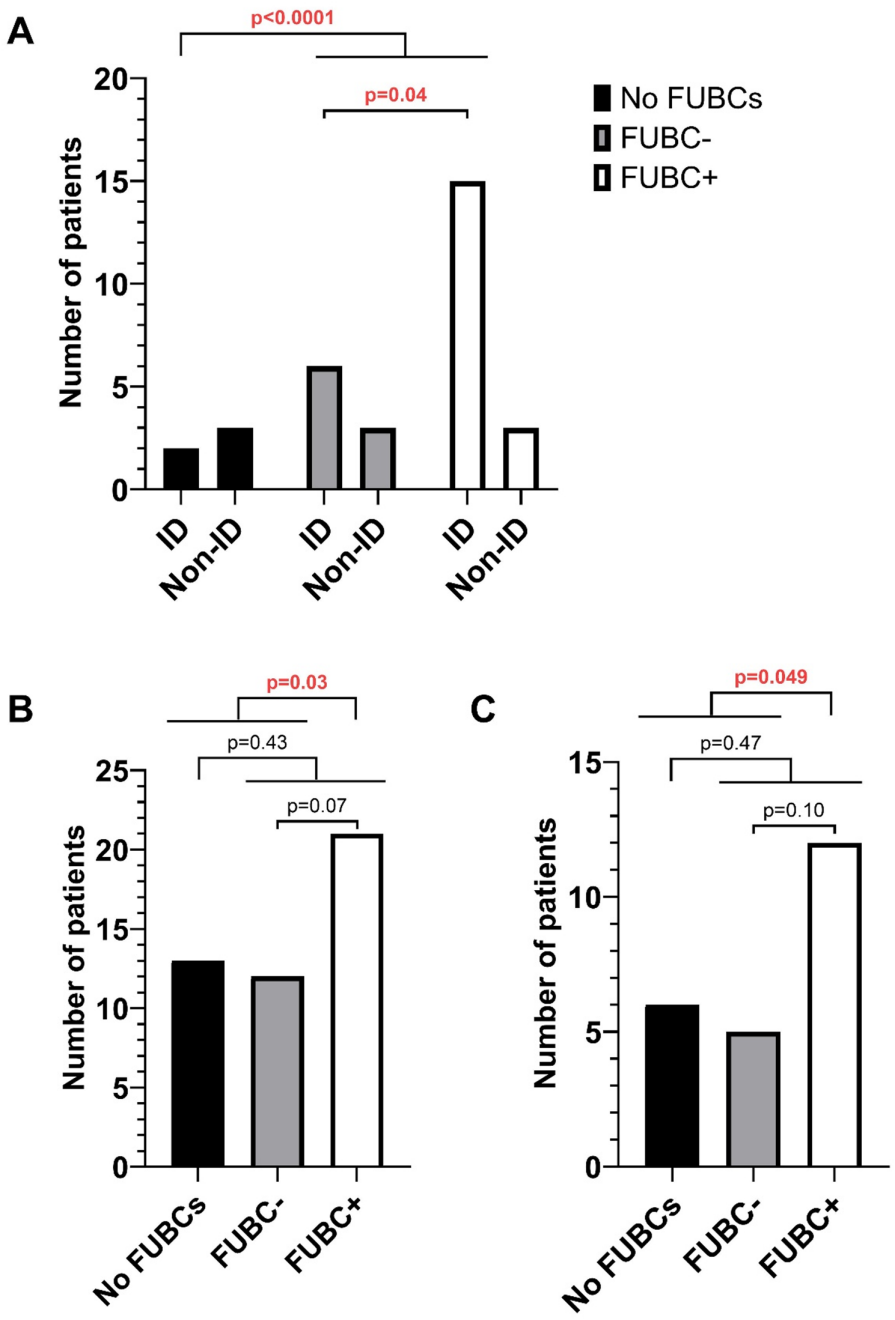
Association of follow-up blood cultures (FUBCs) with variation in clinical care. The number of infection-related consultations, imaging studies, and procedures were identified in matched cohorts of patients with no FUBCs, FUBCs obtained and negative for bacterial growth (FUBC-), and FUBCs obtained and positive for bacterial growth (FUBC+). To understand the potential impact of FUBCs on these infection-related instances, we queried from days 1 through 5 after the FUBC. In patients without a FUBC, we queried from days 1 through 5 after the initial positive blood culture. Within this time window, infection-related consultations (infectious diseases [ID] versus non-ID) (**A**), imaging procedures (**B**), and procedures (**C**) are shown. In (**A**), the p-values are in reference to differences in ID consultations between the groups

Given the observed association and near association between positive FUBCs and increased ID consultation and source control procedures, respectively, we aimed to determine how clinical care differed between patients with positive and negative FUBCs at specific time points. As shown in Fig. 1B, we examined infection-related consultations, imaging studies, and procedures from day 1 through day 5 following the FUBC to identify events that may have been impacted by the FUBC result. As a comparator, we similarly examined such instances from day 1 through day 5 after the initial blood culture in patients without FUBCs. Within this time window, there were 35 infection-related consultations in 32 of the 123 matched patients (32/123 [26%]) (Supplemental Table 4), and the majority were ID consults (23/35 [67%]). The rate of non-ID consults did not differ between the three cohorts, while ID consults were more common in patients with FUBCs generally (No FUBCs: 2/41 [5%]; FUBCs: 21/82 [26%]; *p* < 0.0001) and positive FUBCs in particular (FUBCs negative: 6/41 [15%]; FUBCs positive: 15/41 [37%]; *p* = 0.04) (Fig. 4A).

In this time window, there were 61 infection-related imaging studies in 46 of the 123 matched patients (46/123 [37%]) (Supplemental Table 4). Neither obtaining FUBCs (No FUBCs: 13/41 [32%]; FUBCs: 33/82 [40%]; *p* = 0.43) nor positive FUBCs (FUBCs negative: 12/41 [29%]; FUBCs positive: 21/41 [51%]; *p* = 0.07) were associated with increased number of imaging studies (Fig. 4B). When restricting to imaging studies consistent with infection or possible infection, there was a higher number of imaging studies in patients with positive FUBCs though the association did not meet statistical significance (FUBCs positive: 13/41 [32%]; all others 13/82 [16%]; *p* = 0.06). In this time window, there were 24 infection-related procedures performed with 23 patients (Supplemental Table 4). Positive FUBCs were associated with an increased number of source control procedures (FUBCs positive: 12/41 [29%]; All others: 11/82 [13%]); *p* = 0.049) (Fig. 4C).

To assess links between different infection-related management decisions within this time window, we examined the association between infection-related imaging studies and procedures. Of the 26 patients with imaging findings consistent with infection or possible infection, 9 underwent an infection-related procedure, and they were largely in patients with positive follow-up blood cultures (No FUBCs: 2/9 [22%]; FUBCs negative: 0/9 [0%]; FUBCs positive: 7/9 [78%]).

## Discussion

In this study, we internally validated our earlier findings of decreased mortality with performance of FUBCs in patients with GN-BSI with a more contemporary cohort of patients [5]. Importantly, the association remained significant after accounting for multiple confounding factors, including immortal time bias. We also identified potential mechanisms by which FUBCs might impact clinical care. Each of these is discussed in detail below.

First, this work confirms our earlier findings of decreased mortality in patients with FUBCs and increased mortality in those with persistent GN-BSI. Multiple systematic reviews with meta-analyses showed that obtaining FUBCs were associated with decreased patient mortality [6–8], while a subsequent large study showed no such association [9]. Such variation in findings is not surprising given that the presumed benefit of FUBCs lies in the identification and proper management of patients with persistent GN-BSI, a condition associated with increased risk of relapsed GN-BSI, complications, and death [6, 7, 13–15]. The rate of persistent GN-BSI varies in different patient populations and is higher in those with indwelling prosthetic material, delayed appropriate antibiotic therapy, and certain bacterial species [5, 7]. In this and our previous cohort, persistent GN-BSI was present in ~ 20% of cases. However, in a large Canadian cohort with a high proportion of community-acquired GN-BSI and a lower rate of persistent GN-BSI (11%), FUBCs were associated with decreased mortality in patients with hospital-acquired infections but not in the overall cohort [9]. Differences in analyses and endpoints may also drive variability in the observed relationship between FUBCs and patient mortality. For example, studies that have demonstrated a mortality benefit to FUBCs have generally accounted for patient severity of illness through a marker such as the APACHE-II acute physiology score, Pitt bacteremia score, or SOFA score [5, 16, 17]. In addition, we observed the strongest association between FUBCs and mortality when considering the attributable in-hospital mortality endpoint. By contrast, Ong et al. (2024) measured overall mortality at 30 days. Our current study also validated our earlier finding of increased mortality in patients with persistent GN-BSI [5]. Persistent GN-BSI is a marker for complicated infection and has been consistently associated with increased patient mortality [6, 7].

Second, we identified potential explanations for the observed impact of FUBCs on patient mortality. While we and others have demonstrated an association between FUBCs and patient mortality [5–8], the mechanism of this association is not well understood. A FUBC is a diagnostic test and would not impact patient outcomes by itself. One study demonstrated an association between FUBCs and increased source control [17], suggesting a potential mechanistic link. Here we queried broadly the infection-related consultations, imaging studies, and procedures along with their timing relative to the initial and FUBCs. We found that FUBCs were associated with subsequent ID consultation, a practice associated with decreased mortality in several recent studies [18, 19]. Thus requesting ID consultation may be a consequence of the FUBC returning positive (i.e., persistent GN-BSI). Patients with persistent GN-BSI then also had higher rates of source control procedures. Collectively, positive FUBCs identified patients with a more complicated clinical course associated with more frequent ID consultation, an increased need for source control procedures, and increased mortality.

This work had several limitations. First, it is a single center study and so cannot account for institutional differences in FUBC practices or patient populations. Second, we cannot fully eliminate residual confounding effects from an observational cohort. However, we accounted for selection bias through propensity score-weighted multivariable Cox proportional hazards models, and immortal time bias by treating FUBCs as a time-varying variable and by performing sensitivity analyses to exclude patients that died up to 72 h after the initial positive blood culture. Further, censoring mortality at hospital discharge makes the study susceptible to competing risk bias. Third, the small sample size of the matched cohort likely limited our ability to identify additional statistically significant associations between FUBCs and consultations, imaging studies, and procedures. Finally, our study design does not allow us to identify a causal link between FUBCs and infection-related consultations, imaging, and procedures. However, we believe that by examining the timing of such infection-related instances relative to FUBCs, we can gain insight into how FUBCs may impact patient clinical care.

## Conclusions

We used a contemporary cohort of patients with GN-BSI at our institution to validate our earlier finding of decreased mortality in patients with FUBCs and increased mortality in patients with persistent GN-BSI. These findings remained significant after adjustment for selection bias through an adjusted model, and after adjustment for immortal time bias through multiple approaches. Further, we showed that obtaining FUBCs allowed identification of patients with persistent GN-BSI and were associated with higher rates of infection-related imaging and infectious diseases consultations to identify the source and potential complications of GN-BSI. Positive FUBCs were in turn associated with higher rates of source control and obstruction relieving procedures. Such actions may be part of the mechanistic link between FUBCs and decreased patient mortality.

## Electronic Supplementary Material

Below is the link to the electronic supplementary material.

Supplementary Material 1

## Data Availability

The datasets used and/or analyzed during the current study are available from the corresponding author on reasonable request.

## Abbreviations

FUBC: Follow-up blood culture
GN-BSI: Gram-negative bloodstream infection
IRB: Institutional review board
