# Supplementary material for: The impact of follow-up blood cultures on mortality and management in patients with gram-negative bloodstream infections: a validation cohort study
Source: BMC Infect Dis. 2026 May 28;26:1408. doi: 10.1186/s12879-026-13579-x (PMC13425979; doi:10.1186/s12879-026-13579-x)
Supplement: Supplementary file 1 — Supplementary Material 1 [file 12879_2026_13579_MOESM1_ESM.docx]

**SUPPLEMENTAL METHODS**

*Microbial speciation and susceptibility testing*

All bacterial bloodstream isolates were speciated by the Duke Clinical Microbiology Laboratory using standard techniques. Minimum inhibitory concentration (MIC) values were determined by the Duke Clinical Microbiology Laboratory using either microbroth dilution technique, disk diffusion, or E-test. The antibacterial susceptibility profile of each bacterial isolate, regardless of the year it was isolated, was defined according to the Clinical & Laboratory Standards Institute (CLSI) applicable at the time of the bacteremia episode [1].

Definitions

Bacteremia was categorized as either hospital- or community-acquired [2]. Hospital-acquired bacteremia was defined as bacteremia diagnosed ≥48 hours after hospital admission. Community-acquired bacteremia was defined as the bacteremia diagnosed <48 hours after hospital admission. Community-acquired bloodstream infection was further subdivided into the following: 1) community-acquired, healthcare-associated bloodstream infection, and 2) community-acquired, non-healthcare-associated bloodstream infection. Community-acquired, healthcare-associated bloodstream infection was defined as a bloodstream infection beginning prior to 48 hours after hospital admission in patients that meet one or more of the following criteria: hospitalized in the past 90 days, resident of a nursing home or long-term care facility, actively receiving home intravenous therapy, received wound care or specialized nursing care in previous 30 days, received hemodialysis in past 30 days, immunosuppressed (e.g., presence of metastatic cancer, history of a solid organ or hematological transplant, chemotherapy in last 30 days, currently on immunosuppressive medication for any reason), or surgery in last 180 days. Community-acquired, non-healthcare-associated bloodstream infection is any community-acquired bloodstream infection not meeting the criteria for healthcare-associated bloodstream infection. Source of infection refers to the primary focus of the bacteremia. An endovascular infection included sources such as central venous catheters, etc. Sources that did not fit into a predefined category were placed in “other.” Bacteremia with no single identifiable source was placed in the “none/unknown” category. Cardiac devices are those identified as permanent pacemakers, automatic implantable cardioverter defibrillators, prosthetic heart valves including valvular support rings, and left ventricular assist devices. “Effective antibiotic therapy” is defined as receipt of an antibiotic to which the bacteria are susceptible. “Days to effective therapy” was calculated as the duration in days between the index blood culture (day 0) and effective antibiotic therapy.

**Supplemental Table 1. Characteristics of patients with gram-negative bloodstream infections (GN-BSI) in the matched cohorts.** Patients without follow-up blood cultures (No FUBCs), FUBCs that were obtained and negative for bacterial growth (FUBC-), and FUBCs that were obtained and positive for bacterial growth with the same organisms (FUBC+) were matched (n=41 in each group). Variables that were used to match the cohorts are indicated by an asterisk (*). P-values indicate differences between the three groups and were calculated with either chi-square tests (for categorical variables) or analysis of variance (ANOVA) tests (for continuous variables). Statistically significant differences (p<0.05) are indicated in bold.

| **Variable** | **No FUBCs**  **N=41**  **n (%)** | **FUBC-**  **N=41**  **n (%)** | **FUBC+**  **N=41**  **n (%)** | **P-value** |
| --- | --- | --- | --- | --- |
| **Age (years)*** |  |  |  | 1.00 |
| <50 | 12 (29) | 12 (29) | 12 (29) |  |
| 50-59 | 5 (12) | 5 (12) | 5 (12) |  |
| 60-69 | 10 (24) | 10 (24) | 10 (24) |  |
| 70-79 | 9 (22) | 9 (22) | 9 (22) |  |
| ≥80 | 5 (12) | 5 (12) | 5 (12) |  |
| **Female sex*** | 23 (56) | 22 (54) | 23 (56) | 0.98 |
| **Race** |  |  |  | 0.48 |
| White | 23 (56) | 22 (54) | 17 (41) |  |
| Black | 15 (37) | 18 (44) | 20 (49) |  |
| Other | 3 (7) | 1 (2) | 4 (10) |  |
| **Medical comorbidities** |  |  |  |  |
| IV drug use | 0 | 0 | 0 | 1.00 |
| Corticosteroid use | 7 (17) | 13 (32) | 12 (29) | 0.27 |
| Hematopoietic or solid organ transplant | 2 (5) | 6 (15) | 9 (22) | 0.08 |
| Hemodialysis | 0 | 2 (5) | 2 (5) | 0.36 |
| **Charlson comorbidity index** | 7.6 (4.1) | 8.3 (4.1) | 7.6 (3.7) | 0.63 |
| **Medical devices present** |  |  |  |  |
| Central venous catheter | 2 (5) | 7 (17) | 7 (17) | 0.17 |
| Cardiac device | 2 (5) | 3 (7) | 4 (10) | 0.70 |
| **Source of GN-BSI*** |  |  |  | 1.00 |
| Genitourinary | 31 (76) | 31 (76) | 31 (76) |  |
| Intra-abdominal | 7 (17) | 7 (17) | 7 (17) |  |
| Skin/soft tissue | 0 | 0 | 0 |  |
| Endovascular | 0 | 0 | 0 |  |
| Pulmonary | 0 | 0 | 0 |  |
| Bone/joint | 0 | 0 | 0 |  |
| Other | 1 (2) | 1 (2) | 1 (2) |  |
| None identified | 2 (5) | 2 (5) | 2 (5) |  |
| **Route of GN-BSI*** |  |  |  | 0.99 |
| Hospital-acquired | 5 (12) | 5 (12) | 6 (15) |  |
| Community-acquired, healthcare-associated | 13 (32) | 13 (32) | 12 (29) |  |
| Community-acquired, non-healthcare-associated | 23 (56) | 23 (56) | 23 (56) |  |
| **Hospital service*** |  |  |  |  |
| Medicine | 31 (83) | 31 (83) | 31 (83) | 1.00 |
| Surgery | 3 (7) | 3 (7) | 3 (7) |  |
| Intensive care unit (ICU) | 4 (10) | 4 (10) | 4 (10) |  |
| **Pitt bacteremia score*** |  |  |  | 1.00 |
| 0 | 9 (22) | 9 (22) | 9 (22) |  |
| 1-2 | 24 (59) | 24 (59) | 24 (59) |  |
| 3-4 | 7 (17) | 7 (17) | 7 (17) |  |
| ≥5 | 1 (2) | 1 (2) | 1 (2) |  |
| **Days to effective antibiotic therapy** |  |  |  | 0.27 |
| 0 | 33 (80) | 34 (83) | 29 (71) |  |
| 1 | 7 (17) | 6 (15) | 6 (15) |  |
| 2 | 1 (2) | 1 (2) | 4 (10) |  |
| ≥3 | 0 | 0 | 2 (5) |  |
| **Bacterial group*** |  |  |  |  |
| Enterobacterales | 41 (100) | 41 (100) | 41 (100) | 1.00 |
| Non-Enterobacterales | 0 | 0 | 0 |  |
| **Days from GN-BSI to hospital discharge**  **(mean [standard deviation])** | 5.4 (3.3) | 10.1 (12.9) | 13.0 (12.8) | **0.006** |

**Supplemental Table 2.** Overall infection-related consultations, imaging studies, and procedures in patients with gram-negative bloodstream infections (GN-BSI). Here we show all such instances in matched cohorts of patients with no follow-up blood cultures (FUBCs), FUBCs obtained and negative for growth, and FUBCs obtained and positive for growth. We identified all such instances from the initial positive blood culture through 7 days past either the FUBC (if obtained) or the initial positive blood culture (if no FUBC obtained).

|  | **n (%)** |
| --- | --- |
| **Consultations (n=76)** |  |
| Infectious Diseases | 39 (51) |
| Urology | 19 (25) |
| Gastroenterology | 8 (11) |
| Nephrology | 5 (7) |
| Interventional radiology | 4 (5) |
| General surgery | 2 (3) |
| Orthopaedic surgery | 2 (3) |
| Neurosurgery | 1 (1) |
| Rheumatology | 1 (1) |
| **Imaging studies (n=150)** |  |
| Computed tomography (CT) | 62 (41) |
| X-ray | 36 (24) |
| Ultrasound | 34 (23) |
| Echocardiogram | 10 (7) |
| Magnetic resonance imaging (MRI) | 6 (4) |
| Positron emission tomography (PET) | 1 (1) |
| Hepatobiliary iminodiacetic acid (HIDA) scan | 1 (1) |
| **Procedures (n=38)** |  |
| Percutaneous drain placement | 9 (24) |
| ERCP/cholangioscopy | 6 (16) |
| Line removal | 6 (16) |
| Foley removal | 6 (16) |
| Ureteral stent placement/removal | 5 (13) |
| Incision and drainage | 2 (5) |
| Arthrocentesis | 1 (3) |
| Foley placement | 1 (3) |
| Other hardware removal | 1 (3) |
| Thrombectomy | 1 (3) |

**Supplemental Table 3. Infection-related imaging studies (n=150) in patients with gram-negative bloodstream infection, stratified by whether study showed clear evidence of infection (consistent with infection), possible infection, or no evidence of infection (inconsistent with infection).**

| **Imaging study** | **Consistent**  **with infection**  **n (%)** | **Possible**  **Infection**  **n (%)** | **Inconsistent**  **with infection**  **n (%)** |
| --- | --- | --- | --- |
| Computed tomography (CT) (n=62) | 32 (52) | 11 (18) | 19 (31) |
| X-ray (n=36) | 0 | 13 (36) | 23 (63) |
| Ultrasound (n=34) | 3 (9) | 7 (21) | 24 (71) |
| Echocardiogram (n=10) | 0 | 0 | 10 (100) |
| Magnetic resonance imaging (MRI) (n=6) | 4 (67) | 0 | 2 (33) |
| Positron emission tomography (PET) (n=1) | 0 | 1 (100) | 0 |
| Hepatobiliary iminodiacetic acid (HIDA) scan (n=1) | 0 | 0 | 1 (100) |

**Supplemental Table 4. Infection-related consultations, imaging studies, and procedures in patients with gram-negative bloodstream infections (GN-BSI) within a time window.** Here we show all such instances in matched cohorts of patients with no follow-up blood cultures (FUBCs), FUBCs obtained and negative for growth, and FUBCs obtained and positive for growth. We identified all such instances in a time window from day 1 through day 5 following the FUBC. In the cohort of patients with no FUBCs, we similarly examined such instances from day 1 through day 5 after the initial blood culture in patients without FUBCs.

|  | **n (%)** |
| --- | --- |
| **Consultations (n=35)** |  |
| Infectious Diseases | 23 (67) |
| Urology | 5 (15) |
| Gastroenterology | 5 (15) |
| Nephrology | 1 (3) |
| General surgery | 1 (3) |
| **Imaging studies (n=61)** |  |
| Computed tomography (CT) | 28 (46) |
| Ultrasound | 13 (21) |
| X-ray | 12 (20) |
| Echocardiogram | 6 (10) |
| Magnetic resonance imaging (MRI) | 1 (2) |
| Positron emission tomography (PET) | 1 (2) |
| **Procedures (n=24)** |  |
| ERCP/cholangioscopy | 6 (25) |
| Percutaneous drain placement | 5 (21) |
| Line removal | 4 (17) |
| Foley removal | 3 (13) |
| Ureteral stent placement/removal | 2 (8) |
| Incision and drainage | 2 (8) |
| Arthrocentesis | 1 (4) |
| Foley placement | 1 (4) |

**Supplemental Figure 1. Unadjusted Kaplan-Meier survival curves for patients who did and did not have follow-up blood cultures (FUBCs).**

**
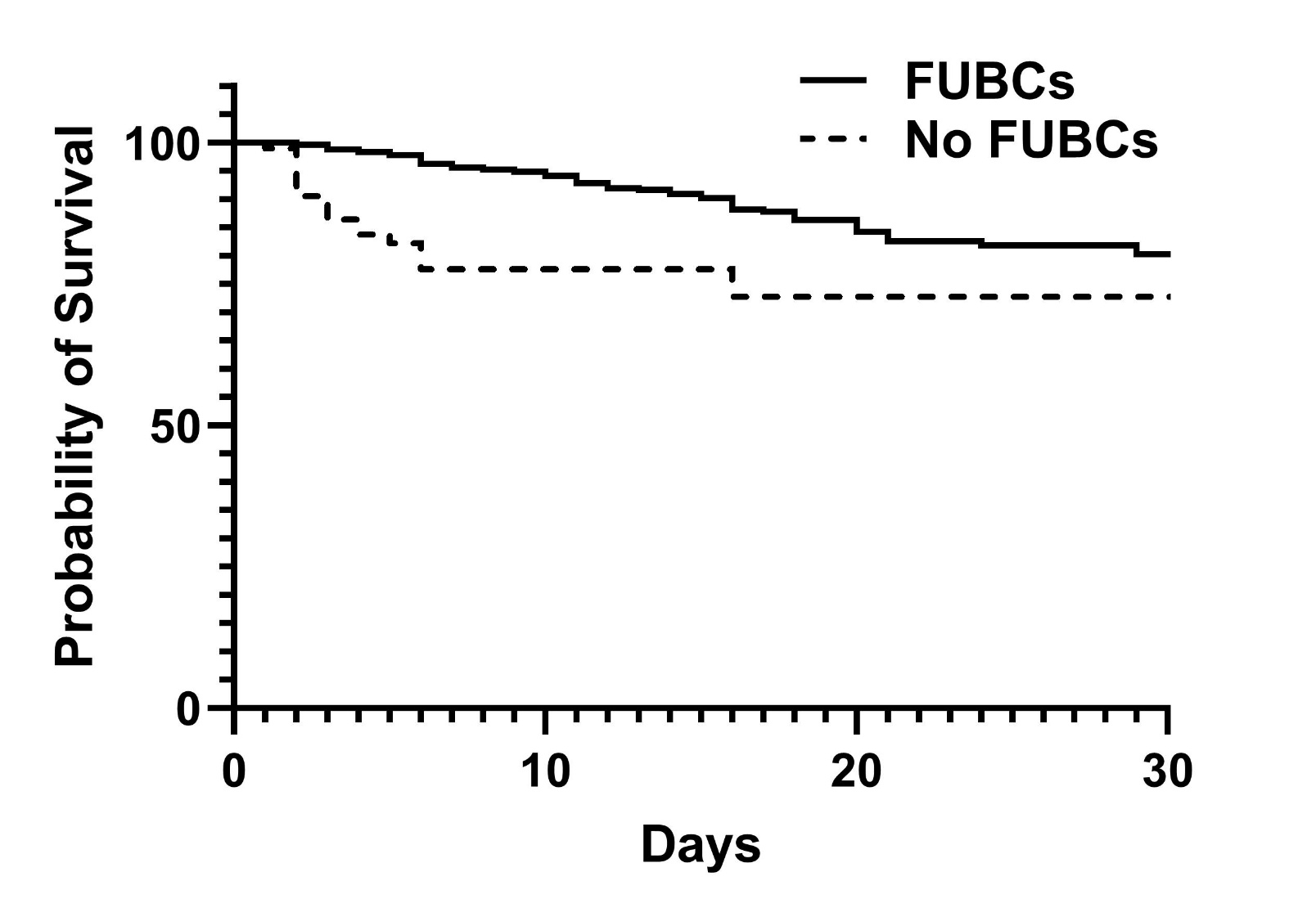
**

**Supplemental Figure 2.** Propensity score-weighted Cox regression model of all-cause in-hospital mortality in patients with gram-negative bloodstream infection. Reference variables are as follows: Bacterial species, *Escherichia* species*;* Hospital service, Medicine; Race, White; Route, Hospital-acquisition; Source, Genitourinary. Variables with p-value less than 0.05 are indicated with an asterisk (*). Abbreviation: FUBCs, follow-up blood cultures.

**
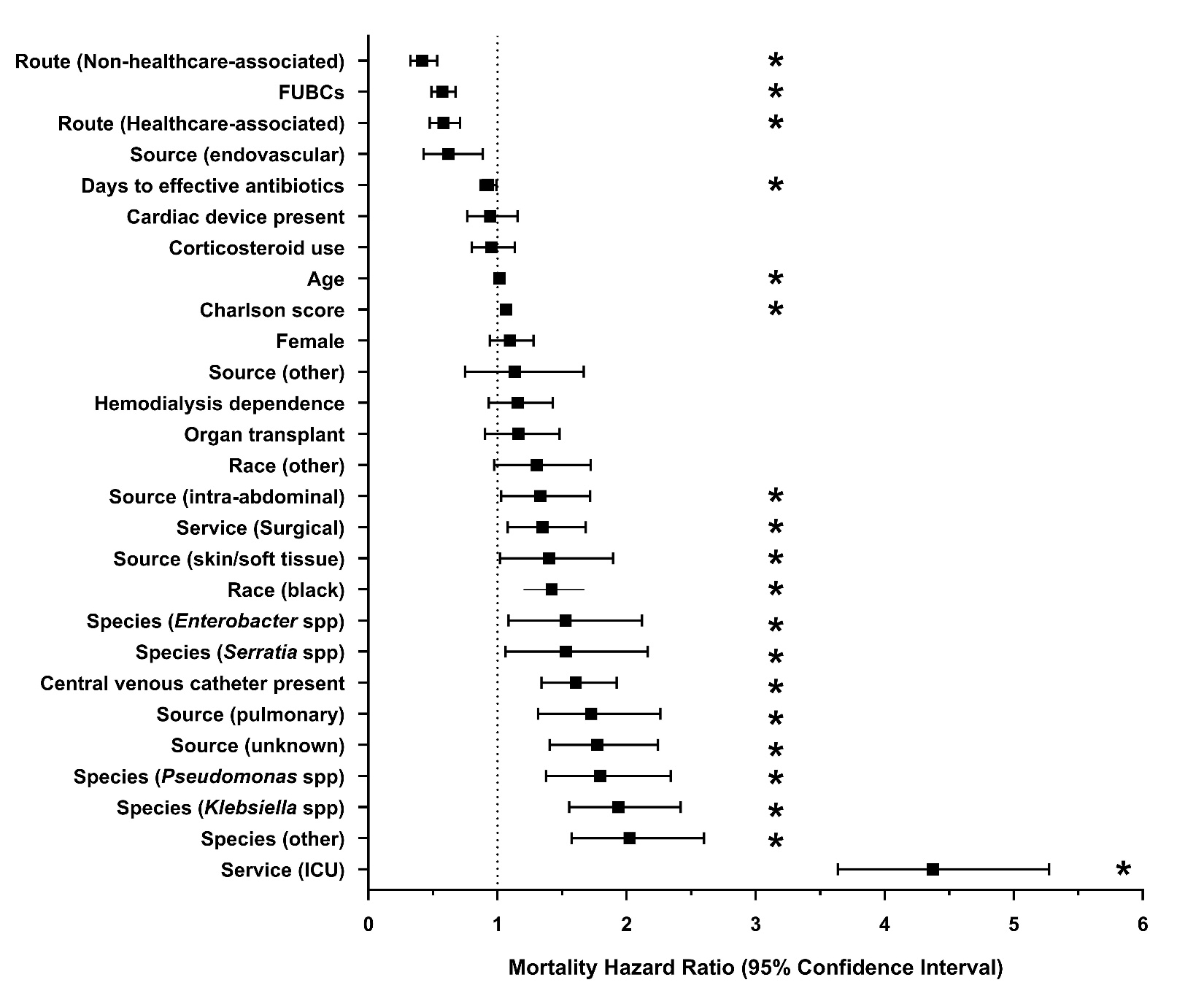
**

**Supplemental Figure 3. Sensitivity analyses to further account for immortal time bias.** Immortal time bias was accounted for by treating follow-up blood cultures (FUBCs) as a time-dependent variable in Cox proportional hazard models and through sensitivity analyses excluding patients that died within 48 hours and 72 hours of the initial positive blood culture (shown here). Multivariable Cox proportional hazards models were generated to assess the impact of FUBCs on attributable and all-cause in-hospital mortality in patients that survived at least 48 hours past the initial positive blood culture (A) and at least 72 hours past the initial positive blood culture (B). The mortality hazard ratio (square) and 95% confidence interval for FUBCs in these models are shown. Associations with p<0.05 are indicated by an asterisk (*).

**
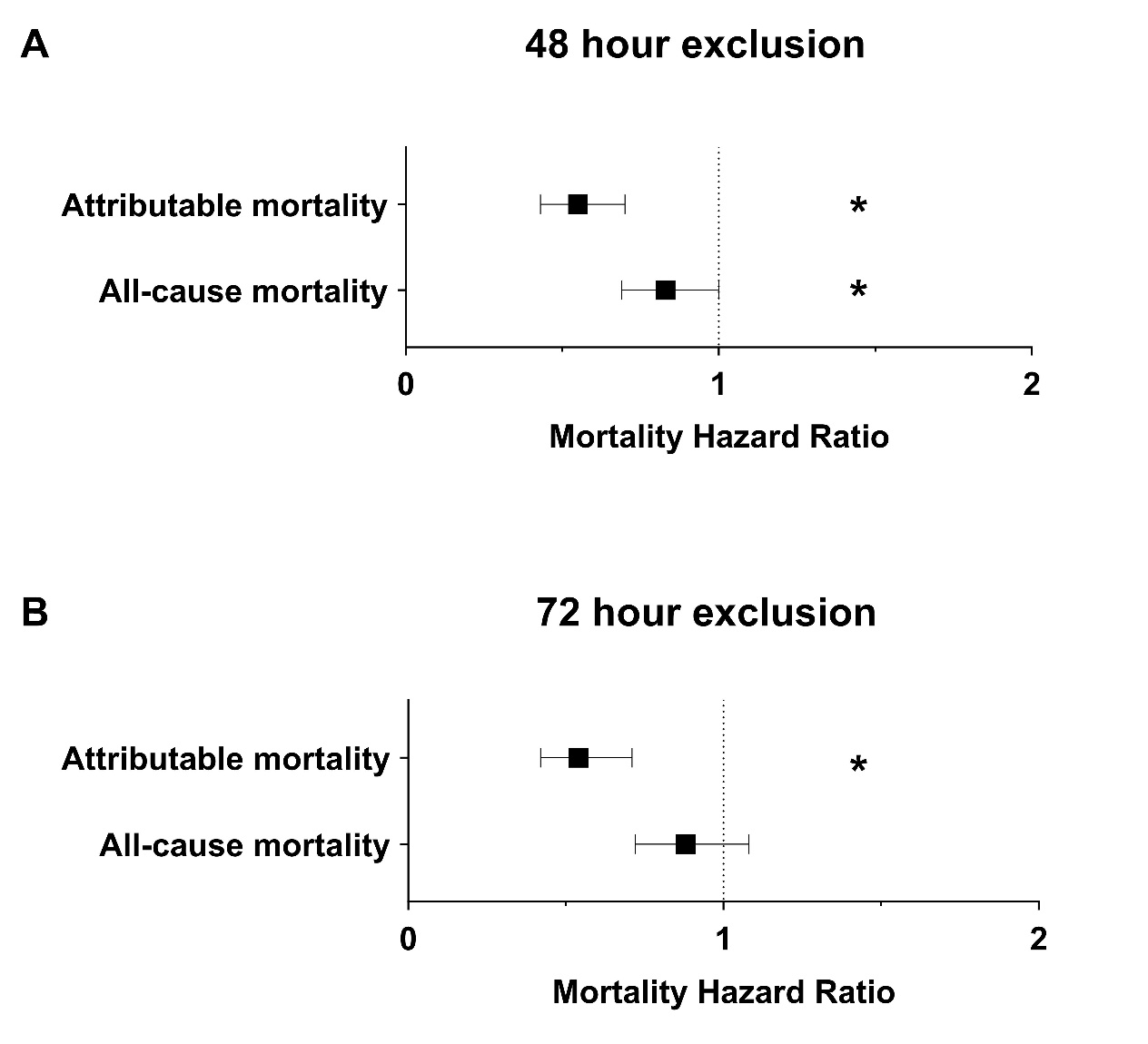
**

**Supplemental Figure 4. Association of persistent gram-negative bloodstream infection (GN-BSI) with mortality.** Propensity score-weighted Cox regression model of all-cause in-hospital mortality in patients with GN-BSI and follow-up blood cultures (FUBCs). FUBCs that were positive for bacterial growth (FUBC+) were not associated with increased total in-hospital mortality relative to FUBCs that were negative for bacterial growth. Reference variables are as follows: Bacterial species, *Escherichia* species*;* Hospital service, Medicine; Race, White; Route, Hospital-acquisition; Source, Genitourinary. Variables with p-value less than 0.05 are indicated with an asterisk (*).

**
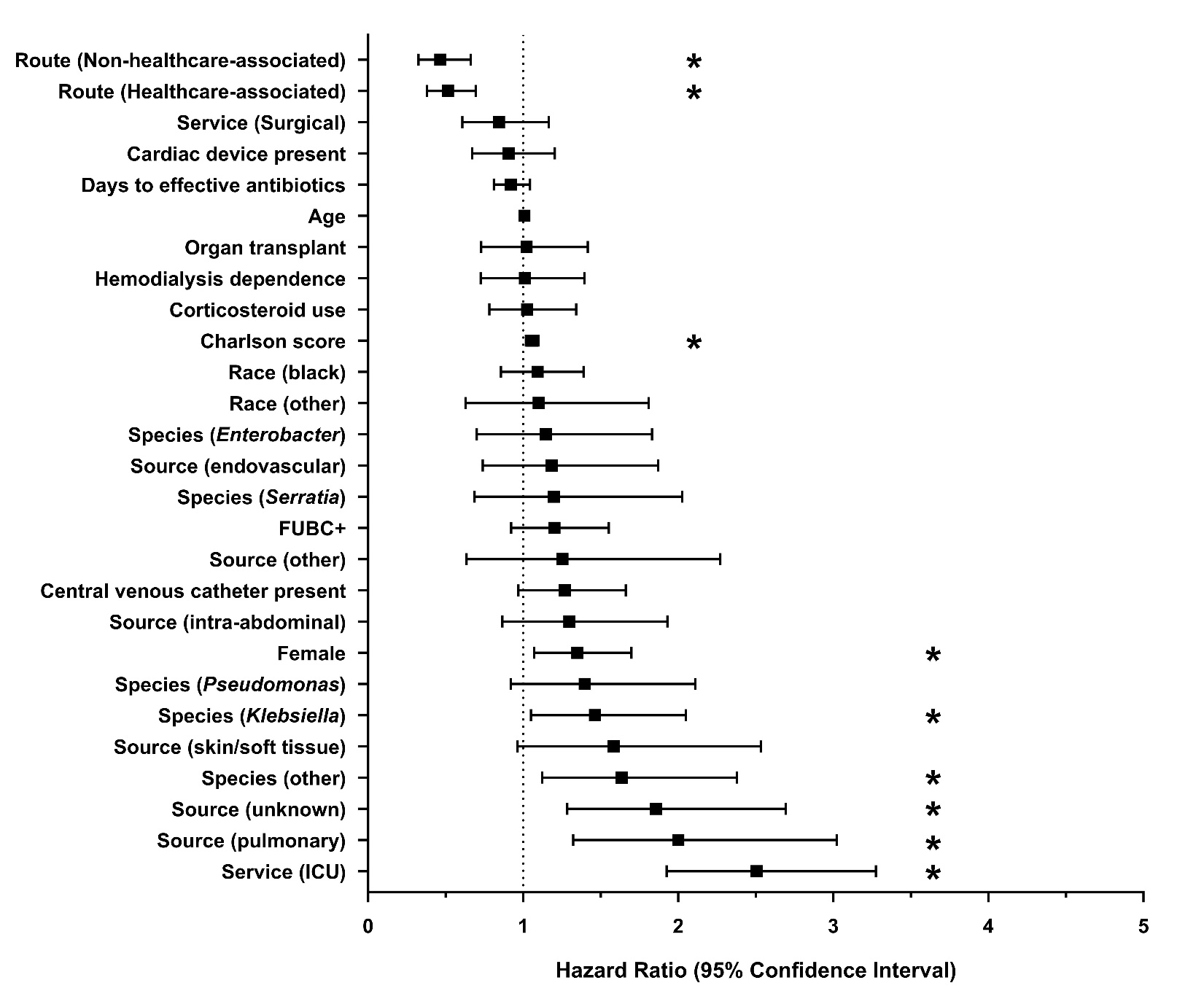
**

**Supplemental Figure 5. Infection-related consultations, imaging studies, and procedures in patients with gram-negative bloodstream infections (GN-BSI), stratified by follow-up blood culture (FUBC) status.** Here we show all such instances in matched cohorts of patients with no follow-up blood cultures (No FUBCs), FUBCs obtained and negative for growth (FUBC-), and FUBCs obtained and positive for growth (FUBC+). We identified all such instances from the initial positive blood culture through 7 days past either the FUBC (if obtained) or the initial positive blood culture (if no FUBC obtained). (A) Infection-related consultations. (B) Infection-related consultations, stratified by whether infectious diseases (ID) or non-ID. (C) Infection-related imaging studies. (D) Infection-related procedures.

**
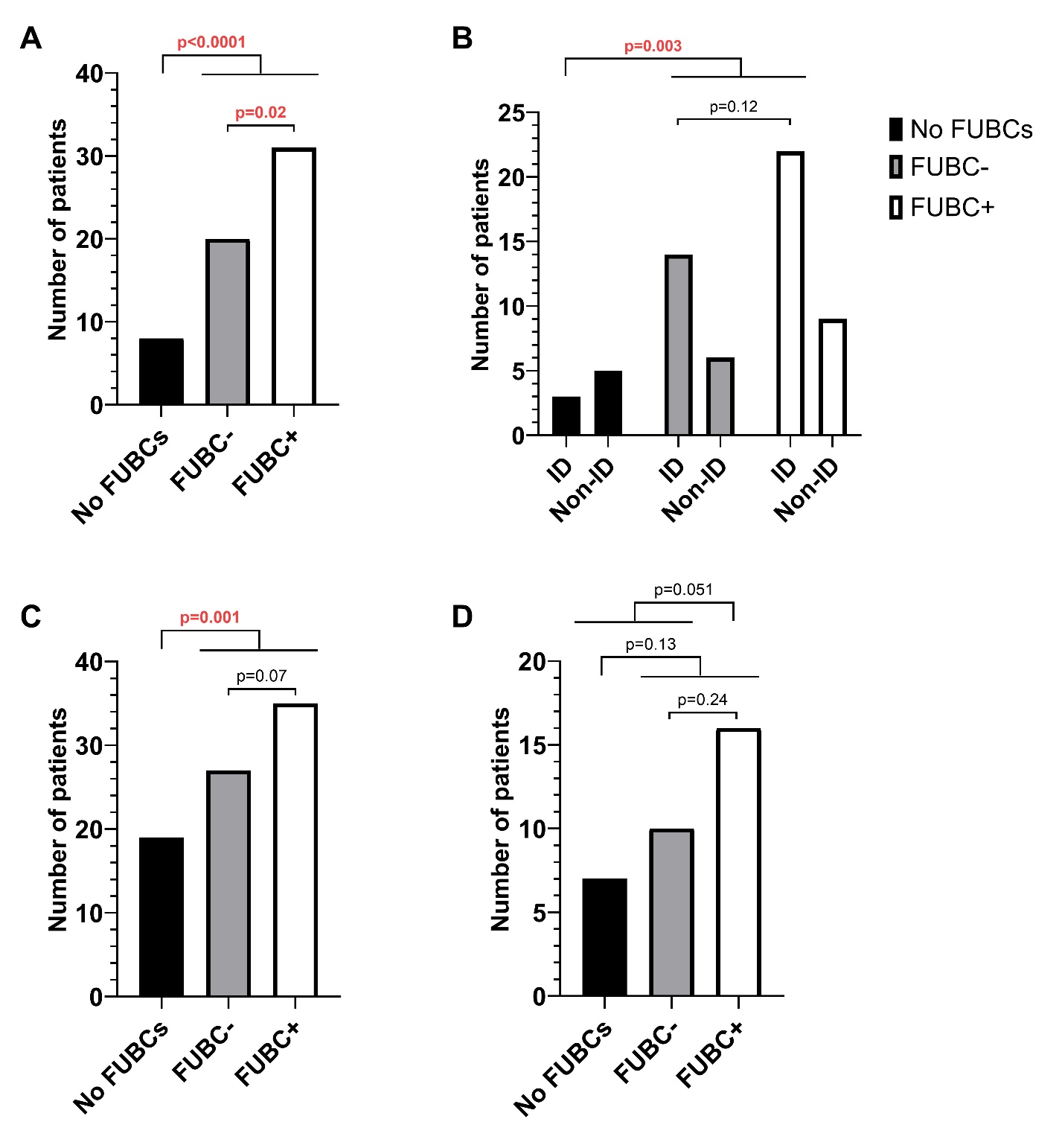
**

**SUPPLEMENTAL REFERENCES**

1. Clinical and Laboratory Standards Institute. *Methods for Dilution Antimicrobial Susceptibility Tests for Bacteria that Grow Aerobically: Tenth Edition M07-A10*. CLSI, Wayne, PA, USA, 2015.

2. Friedman ND, Kaye KS, Stout JE, McGarry SA, Trivette SL, Briggs JP, Lamm W, Clark C, MacFarquhar J, Walton AL, Reller LB, Sexton DJ: Health care--associated bloodstream infections in adults: a reason to change the accepted definition of community-acquired infections. Ann Intern Med. 2002;137(10):791-7.
